# Accessory Breast Carcinoma Masquerading as a Sebaceous Cyst in a Breast Cancer Survivor: A Diagnostic Pitfall in Axillary Assessment

**DOI:** 10.7759/cureus.113541

**Published:** 2026-07-28

**Authors:** Kar Yan Natalie Mok, Poy Wing Lam

**Affiliations:** 1 Department of Radiology, Queen Mary Hospital, Hong Kong, HKG

**Keywords:** accessory breast cancer, accessory breast carcinoma, axillary ectopic breast tissue, axillary mass, breast cancer survivor, delayed diagnosis, ectopic breast tissue

## Abstract

Accessory breast carcinoma is an uncommon malignancy and is often diagnosed late because it could mimic a benign axillary lesion. We report the case of a 63-year-old woman with a prior history of left breast cancer treated with breast-conserving surgery and radiotherapy who later developed a palpable right axillary lump. The lesion had initially been assessed clinically as a sebaceous cyst and was not definitively investigated for 23 months. During routine breast surveillance, mammography and targeted ultrasound revealed a new suspicious lesion arising within accessory axillary breast tissue, which was later proven to be invasive breast carcinoma. Staging investigations, including fluorodeoxyglucose (FDG) PET-CT and contrast-enhanced mammography, showed no synchronous lesions in either orthotopic breast and no evidence of distant metastasis. The patient underwent wide local excision with sentinel lymph node biopsy, followed by adjuvant radiotherapy and hormonal therapy. At seven months’ follow-up, she remained disease-free.

This case underscores the diagnostic difficulty of accessory breast carcinoma and shows how malignancy may be overlooked even during regular breast cancer surveillance. Persistent axillary masses should prompt consideration of accessory breast tissue cancer in all female patients, particularly in those with a history of breast cancer, to enable timely diagnosis and appropriate treatment.

## Introduction

Accessory breast tissue is an embryological remnant along the milk line and is present in approximately 2%-6% of the general population [1]. While breast cancer is one of the most common malignancies among women worldwide, accessory breast carcinoma is an uncommon malignancy, accounting for approximately 0.3%-0.6% of all breast cancers, with the axilla representing the most frequent site because of the predominance of accessory breast tissue in this location [1-2]. The most common presentation is a painless palpable axillary mass, reported in approximately 70%-90% of cases [1-3].

Despite tumor biology closely resembling that of conventional pectoral breast cancer [1-2], diagnosis of accessory breast carcinoma is often delayed. Marshall et al. reported a mean diagnostic delay of 40 months from symptom onset [1]. Consequently, this is thought to contribute to poorer outcomes of accessory breast carcinoma when compared to conventional breast carcinoma [1-3].

We report a case of primary accessory breast carcinoma arising in the axilla of a breast cancer survivor undergoing routine imaging surveillance. The lesion was initially interpreted as a benign sebaceous cyst, resulting in a diagnostic delay of nearly two years. This case highlights the diagnostic challenges associated with malignancy arising in accessory breast tissue, including its rarity, the resulting low index of clinical suspicion, and its nonspecific presentation that may mimic common benign axillary conditions. It also underscores the importance of maintaining an appropriate index of suspicion for persistent or enlarging axillary masses and the need for timely radiological evaluation, clinicopathological correlation, and tissue diagnosis when clinically indicated.

## Case presentation

**Figure 1:**
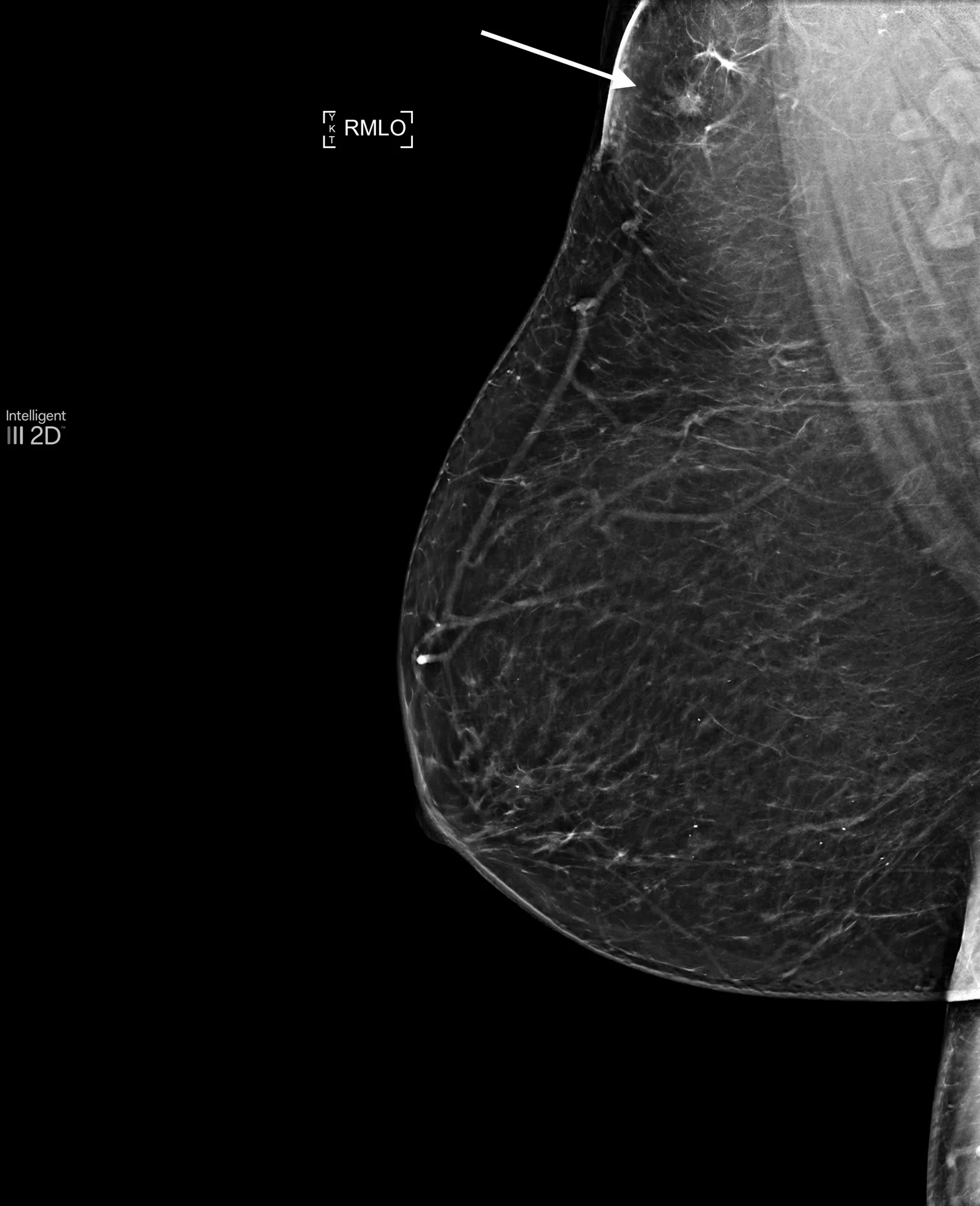
A mammogram during surveillance shows a new irregular spiculated high-density lesion at the right axilla (arrow).

**Figure 2:**
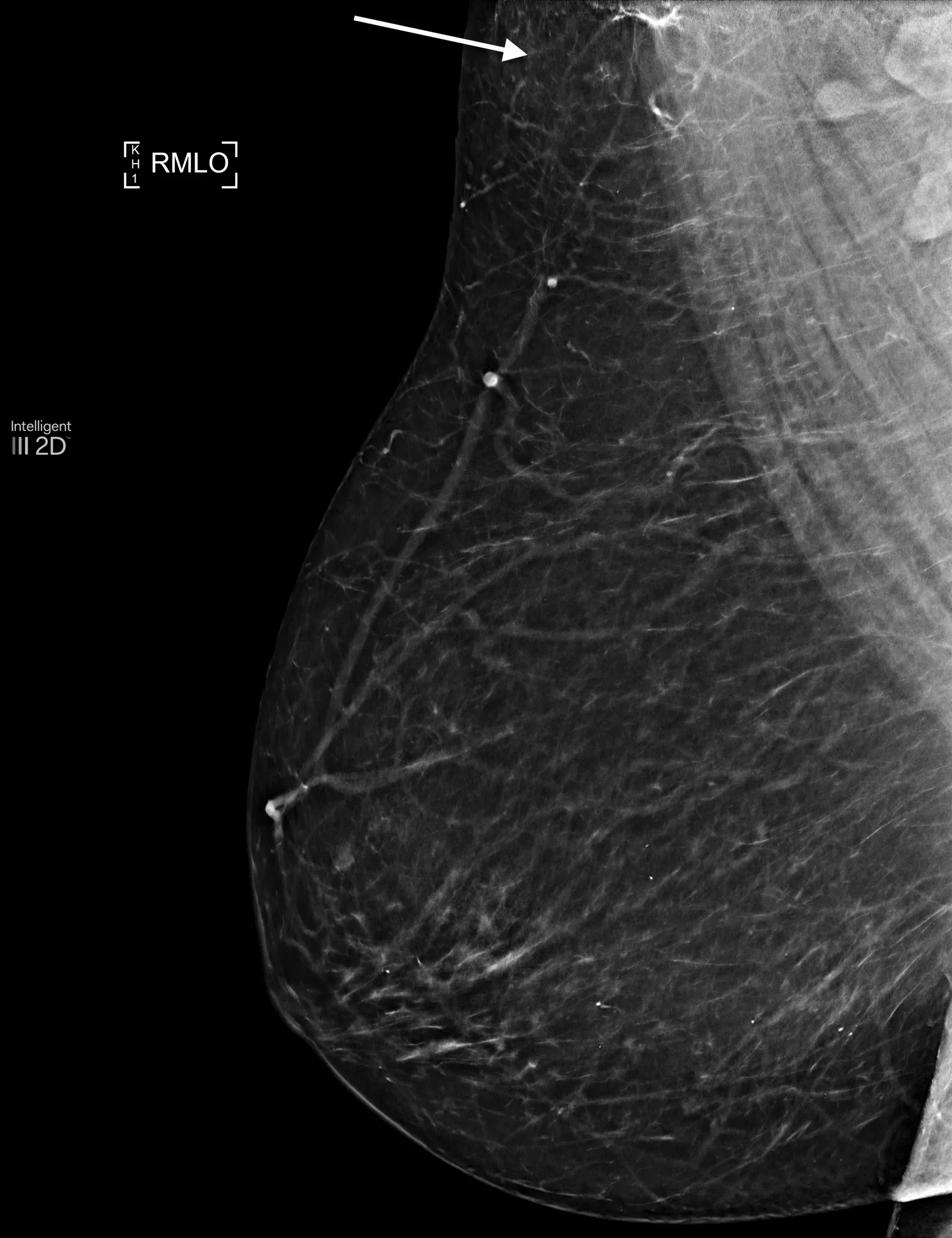
Mammogram 30 months prior shows no such abnormality at the axilla (arrow).

**Figure 3:**
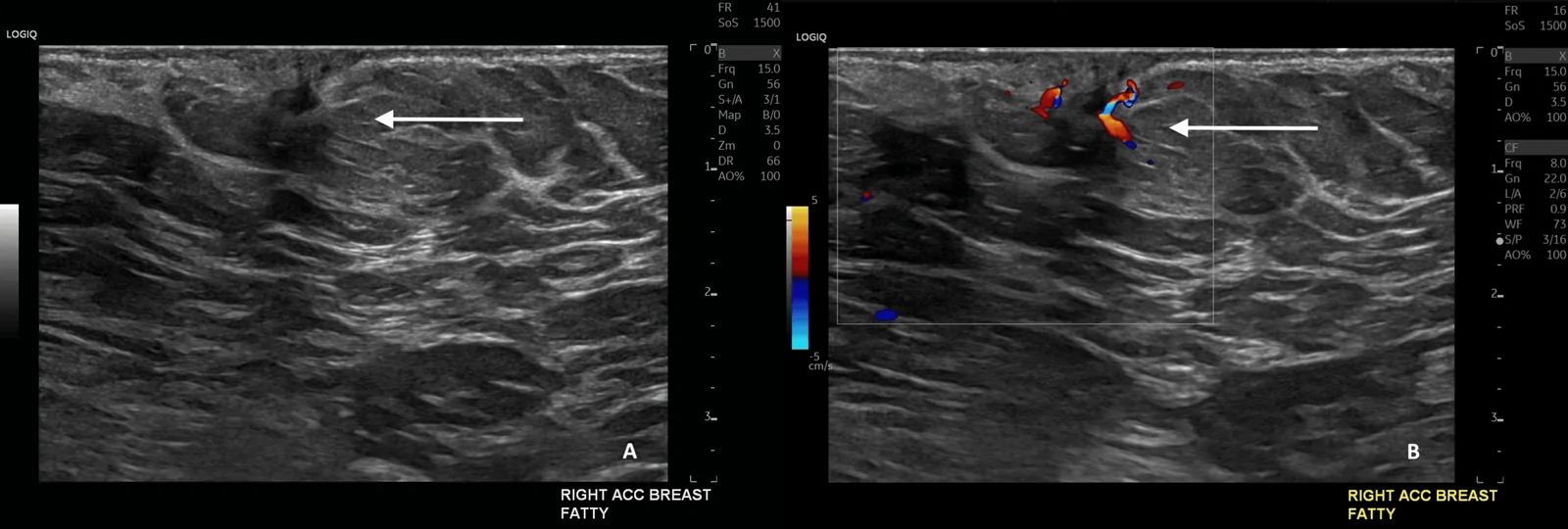
Ultrasound shows a 7mm hypoechoic, irregular spiculated lesion with evidence of skin involvement (A). Colour doppler demonstrates hypervascularity of the lesion (B).

A 63-year-old Indian woman with a history of left intermediate-grade ductal carcinoma in situ of the breast (ER-positive, PR-positive), diagnosed at the age of 55, had been treated with breast-conserving surgery followed by adjuvant radiotherapy and remained under regular breast imaging surveillance thereafter. She initially presented during routine surgical follow-up with a complaint of right axillary swelling that developed following an episode of an upper respiratory tract infection. Clinical examination revealed no palpable cervical or axillary lymphadenopathy. A small, approximately 5 mm palpable lesion was identified within the right axilla, appearing to be attached to the overlying skin with a small dimple that mimics a punctum. Bedside ultrasonography demonstrated a superficial cutaneous lesion that does not correspond to a lymph node and was interpreted as being most consistent with a sebaceous cyst. Surgical excision was offered to obtain a definitive histological diagnosis; however, after discussion of the likely benign clinical and sonographic impression, the patient elected for conservative management. Although the patient continued to notice persistent right axillary swelling, she did not seek further medical attention. Routine breast imaging surveillance was performed 23 months after initial presentation of the axillary lesion. Mammography demonstrated a newly developed high-density irregular spiculated mass in the right axilla (Figures 1-2). A subsequent ultrasound showed that this lesion arose within fatty accessory breast tissue. It was hypoechoic, irregularly spiculated with hypervascularity and evidence of skin involvement (Figure 3). Based on the imaging findings, the leading differential diagnosis was a primary malignancy arising in accessory breast tissue. Other diagnostic considerations included axillary nodal metastasis with extranodal extension, while an inflamed sebaceous cyst was considered less likely given the lesion's suspicious imaging characteristics.

Histopathological examination of the ultrasound-guided core biopsy demonstrated an invasive epithelial malignancy infiltrating fibroadipose tissue. Immunohistochemistry showed diffuse GATA3 positivity and preserved membranous E-cadherin expression, supporting breast epithelial differentiation and a ductal phenotype. The tumor demonstrated strong hormone receptor expression (ER-positive, PR-positive) and was HER2-negative. These findings were consistent with invasive carcinoma of no special type (NST).

**Figure 4:**
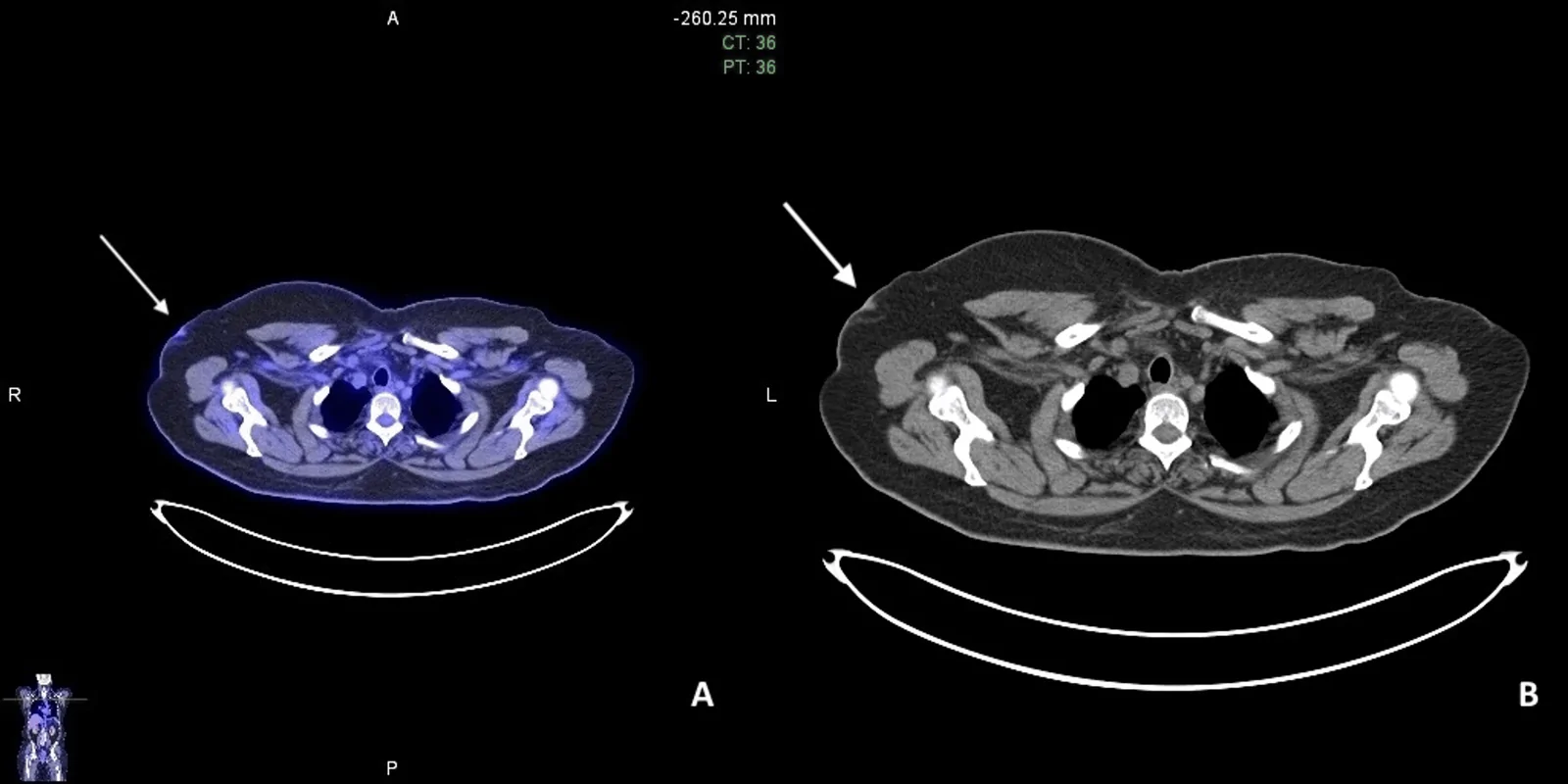
PET-CT shows only mild metabolic activity within the right axilla lesion (A and B, maximum standardized uptake value (SUVmax) 2.7), with no evidence of synchronous lesion within either orthotopic breast, regional nodal involvement or distant metastatic disease.

Staging fluorodeoxyglucose (FDG) PET-CT demonstrated only mild metabolic activity within the lesion (maximum standardized uptake value (SUVmax) 2.7) (Figure 4), with no evidence of malignancy within either orthotopic breast, regional nodal involvement, or distant metastatic disease. Contrast-enhanced mammography likewise demonstrated no synchronous lesion within either orthotopic breast. Correlation of the pathological findings with the lesion's radiological localization within accessory breast tissue and the absence of an occult orthotopic primary tumor or metastatic disease established the diagnosis of a primary accessory breast carcinoma. 

Treatment

Following multidisciplinary discussion, management was undertaken according to contemporary principles for conventional breast cancer. The patient underwent wide local excision of the accessory breast malignancy with sentinel lymph node biopsy, followed by adjuvant radiotherapy and hormonal therapy with a planned treatment duration of at least five years. Histopathological examination demonstrated a 7.7 mm invasive carcinoma of NST, low proliferative index (Ki-67 <10%), ER score 7, PR score 4, HER2-negative status, with clear surgical margins and negative sentinel lymph nodes. Final pathological staging was pT1bN0. 

Outcome and follow-up

The patient remains under joint follow-up by breast surgeons and clinical oncologists. At seven months postoperatively, she was clinically disease-free with no evidence of local recurrence or distant metastasis. Treatment-related morbidity was limited to mild right upper-limb lymphoedema and post-radiotherapy skin changes. Germline BRCA testing was negative. Ongoing surveillance includes annual mammographic assessment.

## Discussion

Accessory breast tissue, also known as ectopic or supernumerary breast tissue, results from incomplete regression of the embryological mammary ridge (milk line), which extends bilaterally from the axilla to the groin. It is present in approximately 2%-6% of the general population, with the axilla representing the most common location [1, 2]. Similar to orthotopic breast tissue, accessory breast tissue is susceptible to physiological changes and may develop a spectrum of benign and malignant conditions, including fibroadenoma, fibrocystic change, mastitis, phyllodes tumor, and primary breast carcinoma [2].

Primary accessory breast tissue carcinoma is rare, accounting for only 0.3%-0.6% of all breast cancers [1-2]. Histologically, invasive carcinoma of NST is the most frequently reported subtype, with tumor biology closely resembling that of conventional pectoral breast cancer [1-2]. The clinical presentation of accessory breast tissue carcinoma is often nonspecific, which contributes to delayed diagnosis. The most common presentation is a painless palpable axillary mass, reported in approximately 70%-90% of cases [1-3], as the axilla is the most common location of accessory breast tissue. Patients with more advanced disease may present with associated skin changes [1-2] and/or palpable axillary lymph nodes [3]. 

The rarity of the disease and the frequent failure to recognize accessory breast tissue contribute to a low index of clinical suspicion and consequently delay diagnosis [1-2]. In this case, the lesion had previously been attributed to a benign sebaceous cyst without histological confirmation, resulting in a delay of nearly two years before definitive diagnosis. Similar diagnostic delays have been repeatedly reported in the literature, where these axillary lesions are mistaken for benign conditions such as sebaceous cysts, lipomas, reactive lymphadenopathy, or metastatic axillary nodes from an occult primary breast lesion [1, 2, 4, 5]. In addition, accessory breast tissue may lie outside the routine field of clinical examination and standard mammographic coverage, particularly when located outside the axilla, which may further contribute to delayed recognition and diagnostic difficulty [5, 6].

In a classic review of ectopic breast carcinoma published in Surgical Oncology, Marshall et al. reported a mean delay of 40 months from symptom onset to diagnosis [1]. This prolonged interval is clinically relevant because delayed recognition may contribute to a more advanced stage at presentation and potentially more complex treatment. Zhang et al. reported that 81.8% of patients presented with stage II or higher disease, 45.5% had axillary lymph node metastases, and 36.4% had distant metastases at diagnosis. Although limited by its small sample size, these findings are consistent with earlier reports suggesting that delayed recognition contributes to presentation at a relatively advanced stage [3].

At present, no dedicated international guidelines address the screening or surveillance of accessory breast tissue. Nevertheless, this case underscores the importance of maintaining awareness of accessory breast tissue during routine clinical assessment and breast imaging. Clinicians and radiologists should recognize that accessory breast tissue is susceptible to the same spectrum of benign and malignant pathologies as orthotopic breast tissue and should therefore interpret persistent or suspicious axillary abnormalities with an appropriate index of suspicion. In patients with known accessory breast tissue, persistent or enlarging lesions should prompt careful clinical evaluation, targeted imaging, and tissue sampling when indicated. Greater awareness of the potential locations and imaging appearances of accessory breast tissue may facilitate earlier diagnosis and appropriate management of this uncommon malignancy [2, 5, 6].

The present case is also notable because accessory breast tissue can serve as the site of a second primary malignancy in patients with a prior history of breast cancer [4]. While accessory breast carcinoma should be considered in all patients presenting with a persistent axillary mass, it is equally important to distinguish a primary tumor arising in accessory breast tissue from recurrent breast cancer or nodal metastasis via appropriate clinicopathological correlation, as this distinction has important implications for staging and management [2, 4]. Prognosis in accessory breast carcinoma appears to be determined primarily by tumor stage, nodal status, and receptor biology, similar to conventional breast cancer [1-3]. The poorer outcomes reported historically are believed to result predominantly from delayed diagnosis, leading to larger primary tumors and more frequent nodal involvement at presentation, rather than from intrinsically more aggressive tumor biology [1-2].

Although no dedicated international management guidelines currently exist for primary accessory breast carcinoma, most authors recommend management according to established principles for orthotopic breast cancer. This includes complete surgical excision with histologically negative margins, appropriate axillary staging, and the use of adjuvant radiotherapy and systemic therapy guided by tumor stage, receptor status, and tumor biology [2, 7]. The favorable outcome observed in the present case supports the applicability of this treatment approach when the disease is diagnosed at an early stage, before the development of regional nodal or distant metastatic disease.

## Conclusions

This case highlights the diagnostic challenges associated with accessory breast carcinoma, including its rarity, the resulting low index of clinical suspicion, and its nonspecific presentation that may mimic common benign axillary conditions, and also demonstrates how malignancy arising in ectopic breast tissue may be overlooked, even in patients undergoing regular breast cancer surveillance. Persistent or enlarging axillary masses warrant careful evaluation and consideration of accessory breast tissue pathology, particularly in individuals with a personal history of breast cancer. Increased awareness among clinicians and radiologists may facilitate earlier diagnosis and timely treatment of this rare but clinically significant disease.
